# Characterization and Comparative Analysis of RWP-RK Proteins from *Arachis duranensis*, *Arachis ipaensis*, and *Arachis hypogaea*

**DOI:** 10.1155/2020/2568640

**Published:** 2020-08-27

**Authors:** Chenyang Liu, Dongliang Yuan, Tong Liu, Mengge Xing, Wenying Xu, Huiying Zhang, Hanqi Jin, Chunmei Cai, Shuai Li

**Affiliations:** College of Life Sciences, Key Lab of Plant Biotechnology in Universities of Shandong Province, Qingdao Agricultural University, Qingdao, 266109 Shandong Province, China

## Abstract

RWP-RK proteins are important factors involved in nitrate response and gametophyte development in plants, and the functions of RWP-RK proteins have been analyzed in many species. However, the characterization of peanut RWP-RK proteins is limited. In this study, we identified 16, 19, and 32 *RWP-RK* members from *Arachis duranensis*, *Arachis ipaensis*, and *Arachis hypogaea*, respectively, and investigated their evolution relationships. The RWP-RK proteins were classified into two groups, RWP-RK domain proteins and NODULE-INCEPTION-like proteins. Chromosomal distributions, gene structures, and conserved motifs of *RWP-RK* genes were compared among wild and cultivated peanuts. In addition, we identified 12 orthologous gene pairs from the two wild peanut species, 13 from *A. duranensis* and *A. hypogaea*, and 13 from *A. ipaensis* and *A. hypogaea*. One, one, and seventeen duplicated gene pairs were identified within the *A. duranensis*, *A. ipaensis*, and *A. hypogaea* genomes, respectively. Moreover, different numbers of *cis*-acting elements in the *RWP-RK* promoters were found in wild and cultivated species (87 in *A. duranensis*, 89 in *A. ipaensis*, and 92 in *A. hypogaea*), and as a result, many *RWP-RK* genes showed distinct expression patterns in different tissues. Our study will provide useful information for further functional and evolutionary analysis of the *RWP-RK* genes.

## 1. Introduction

Transcription factors are essential components of plant signal transduction pathways. The *Arabidopsis* genome contains approximately 1500 transcription factors distributed among various signaling pathways [1]. To transfer a signal to its target genes, a transcription factor binds to *cis*-acting elements in the genes' promoter regions to activate or suppress their expression. Some transcription factors contain similar motifs, have similar functions, and are classified in the same gene family, while others with different conserved domains are placed in different gene families. Many plant transcription factor gene families have been identified and characterized, such as the SQUAMOSA promoter binding protein (SBP) box family, the heat shock transcription factor (Hsf) family, and the RWPXRK motif (RWP-RK) gene family [2–8].

The *RWP-RK* gene family is characterized by a conserved motif, a 60 amino acid sequence that is thought to be involved in DNA binding, and has been studied in many species [4, 9, 10]. For example, there are 14, 8, and 15 *RWP-RK* members in *Arabidopsis*, *Medicago truncatula*, and *Oriza sativa*, respectively. Moreover, the RWP-RK proteins are classified into two groups, the RWP-RK domain proteins (RKD) and the NODULE-INCEPTION-like proteins (NLP), on the basis of their gene structures. In addition to the RWP-RK motif, all NLPs contain a PB1 (Phox and Bem1) motif, and some members contain a GAF (a new class of cyclic GMP receptor) or GAF-like domain [4, 11, 12]. The PB1 domain is thought to be involved in protein-protein interactions, and the GAF domain participates in signal transduction or dimerization in plants [4, 13, 14].

The functions of several RWP-RK proteins have been investigated in the past decades [4]. For example, *NLP* genes have been shown to function in the plant nitrate response. In *Arabidopsis*, *AtNLP6* and its closest homolog, *AtNLP7*, are thought to regulate the N starvation response by binding to N metabolism genes and downstream regulatory genes [4, 15, 16]. In maize, *ZmNLP3.1*, *ZmNLP6*, and *ZmNLP8* rescue *Arabidopsis nlp7* phenotypes and are involved in primary nitrate response by regulating nitrate assimilation under low nitrate conditions [16]. Another *NLP* gene, *NIN*, is a key factor in legume nodule formation [4]. *NIN* was the first transcription factor identified as functioning in Lotus japonicus nodulation, and mutation of *L. japonicus NIN* blocks infection and prevents nodule organogenesis [17]. In *M. truncatula*, *MtNIN* plays a central role in the temporal and spatial regulation of the nodule development process by competitively inhibiting *ERF required for nodulation* (*ERN1*) to suppress the expression of *Early Nodulin 11* (*ENOD11*) and increase the expression of *Cytokinin Response 1* (*CRE1*) [4, 16]. Moreover, soybean *GmNINa*, the ortholog of *L. japonicus NIN*, regulates nodulation through interactions with *Nodule Number Control 1* (*NNC1*) and the activation of *miR172c* [18].

Many RKD proteins have been shown to function in the gametophyte development [4, 19]. The *Arabidopsis* genome contains five *RKD* members, designated *AtRKD1* to *AtRKD5*. The expression of *AtRKD1*, *AtRKD2*, *AtRKD3*, and *AtRKD4* is high in reproductive organs, whereas the expression of *AtRKD5* is ubiquitous [20, 21]. *AtRKD1* and *AtRKD2* have overlapping functions during plant development, and constitutive expression of *AtRKD1* or *AtRKD2* in sporophyte cells alters gene transcription patterns, causing them to resemble those of egg cells [20]. The *rkd4* mutant shows impaired zygotic cell elongation [22], and *RKD3* and *RKD4* are involved in ensuring polarity and specifying cell identity in the female gametophyte [19].

Peanut is an important legume crop that provides oil and food worldwide. The cultivated peanut, *A. hypogaea* (AABB genome, 2*n* = 4*x* = 40), is thought to be descended from the hybridization and polyploidization of two wild diploids: *A. duranensis* (AA genome, 2*n* = 2*x* = 20) and *A. ipaensis* (BB genome, 2*n* = 2*x* = 20) [23–25]. Genome size and gene content have changed during the evolution of the cultivated peanut, and polyploidization has produced many duplicated gene pairs [25–29]. Some duplicated genes have evolved new functions, and some have become pseudogenes [30, 31]. The RWP-RK proteins have also changed during evolution, and research on their function in nitrate response and gametophyte development has been carried out in many plant species [4]. However, information on peanut RWP-RK proteins is limited. In this study, we identified and characterized *RWP-RK* members from wild and cultivated peanuts and investigated the evolutionary relationships among them. Our research will provide essential information for further functional characterization and peanut improvement.

## 2. Materials and Methods

### 2.1. Identification of *RWP-RK* Genes from Wild and Cultivated Peanut Genomes

The amino acid sequences of the conserved RWP-RK domain (PF02042) and *Arabidopsis* RWP-RK proteins downloaded from TAIR (https://www.arabidopsis.org/) were used as BLAST queries against the peanut genome database (https://www.peanutbase.org/) to search for wild and cultivated peanut *RWP-RK* genes. We also searched for *RWP-RK* candidate genes using gene annotations in the peanut genome database. The conserved RWP-RK domains in these candidate *RWP-RK* genes were confirmed by Pfam (http://pfam.xfam.org/search) and NCBI (National Center for Biotechnology Information). The genomic length, CDS length, and number of amino acids for each gene were obtained from the peanut genome database. The characteristics of the peanut *RWP-RK* genes, including their molecular weights and theoretical isoelectric points, were predicted using ProtParam (https://web.expasy.org/protparam/), and their GC content was determined using DNASTAR (DNASTAR, Madison, WI, USA) [32].

### 2.2. Phylogenetic Relationship Analysis

The amino acid sequences of the RWP-RK proteins from *Arabidopsis*, *A. duranensis*, *A. ipaensis*, *A. hypogaea*, *M. truncatula*, and other plant species described by Chardin et al. [4] were aligned using ClustalX2 [33]. The resulting alignments were used to construct a phylogenetic tree in MEGA 7.0 with the neighbor-joining method [34]. The RWP-RK proteins from *A. duranensis*, *A. ipaensis*, and *A. hypogaea* were also used to construct a phylogenetic tree in MEGA 7.0 with the neighbor-joining method.

### 2.3. Gene Structure and Conserved Motif Analyses

The genomic and CDS sequences of the wild and cultivated peanut *RWP-RK* genes were obtained from the peanut genome database and used to construct exon-intron organization maps with the Gene Structure Display Server program [35]. The full lengths of the RWP-RK proteins were used for conserved motif analysis with MEME tools (http://meme-suite.org/), and the positions of the conserved RWP-RK and PB1 domains in each gene were determined with Pfam.

### 2.4. Analysis of *cis*-Acting Elements in Promoter Regions

The promoter region of each *RWP-RK* gene from *A. duranensis*, *A. ipaensis*, and *A. hypogaea*, 2 kb upstream of the initiation codon, was downloaded from the peanut genome database. *cis*-Acting elements in these promoter regions were investigated using PlantCARE (http://bioinformatics.psb.ugent.be/webtools/plantcare/html/) [36] and categorized based on their putative functions.

### 2.5. Chromosomal Locations, Orthologous Gene Pairs, and Gene Duplication Analyses

The physical positions of *RWP-RK* members were obtained from the peanut genome database, and chromosomal location maps were generated using MapInspect software (http://www.mybiosoftware.com/mapinspect-compare-display-linkage-maps.html). The evolutionary relationships of orthologous gene pairs were assessed based on phylogenetic trees [37]. The *RWP-RK* genes of *A. duranensis*, *A. ipaensis*, and *A. hypogaea* were clustered using OrthoMCL software (https://orthomcl.org/orthomcl/) to analyze duplicated gene pairs, and the duplicated gene pairs were drawn using Circos software [5, 38–40].

### 2.6. Transcription Pattern Analysis of the *RWP-RK* Genes

RNA-seq datasets were downloaded from the peanut genome database (https://peanutbase.org/gene_expression) [41]. Twenty-two different tissues from the cultivated peanut, *A. hypogaea*, were collected as described by Clevenger et al. [41]. These samples were used for gene expression analysis. The datasets of the *A. hypogaea* gene expression mapped to *A. duranensis* and *A. ipaensis* were used to investigate *RWP-RK* expression levels in wild peanuts, and a heatmap was constructed with Multiple Experiment Viewer 4.9.0 [42]. Colors in the heatmap indicate the FPKM values of the genes. To validate these gene expressions, tissues, described by Jin et al. [37], were sampled from *A. duranensis* PI219823, *A. ipaensis* PI468322, and *A. hypogaea* Tiffrunner grown in the field in Qingdao, China. For the N treatment, hydroponic experiments were performed using the culture solution as described by Li et al. [43], and 10-day-old peanut plants were grown in normal and N-free solution culture. The whole plants were collected for gene expression analysis. Each sample was analyzed using three biological replicates. RNA extraction, quantitative real-time PCR (qRT-PCR), and expression analysis were performed as described by Jin et al. [37]. All the primers used are listed in Table S1.

## 3. Results

### 3.1. Identification of RWP-RK Proteins in Wild and Cultivated Peanuts

To obtain an exhaustive list of the RWP-RK proteins from wild and cultivated peanuts, we used gene annotations and BLAST searches of the peanut genome database. We found 17, 27, and 37 RWP-RK proteins in *A. duranensis*, *A. ipaensis*, and *A. hypogaea*, respectively, using gene annotations. We also used the amino acid sequences of the conserved RWP-RK domain (PF02042) and of 14 RWP-RK proteins from Arabidopsis as BLAST queries, and we found 18, 21, and 34 RWP-RK proteins in *A. duranensis*, *A. ipaensis*, and *A. hypogaea*, respectively, using this method. We used Pfam to determine whether each candidate gene contained the conserved RWP-RK domain, and we ultimately confirmed the presence of 16, 19, and 32 *RWP-RK* members in *A. duranensis*, *A. ipaensis*, and *A. hypogaea*, respectively (Tables 1 and 2). The genomic length, CDS length, and amino acid number of the *RWP-RK* genes differed among wild and cultivated peanuts. In *A. duranensis*, the genomic length ranged from 779 (*Aradu.1V6B6*) to 13327 bp (*Aradu.G4SB3*), the CDS length varied from 180 (*Aradu.I1BME*) to 4212 bp (*Aradu.G4SB3*), and the number of amino acid residues ranged from 59 to 1403. In *A. ipaensis*, the genomic length ranged from 771 (*Araip.KR88K*) to 10861 bp (*Araip.73BCB*), the CDS length ranged from 309 (*Araip.KR88K*) to 2955 bp (*Araip.R44NW*), and the number of amino acid residues varied from 102 to 984. By contrast, in the cultivated peanut A. hypogaea, the genomic length ranged from 291 (*Arahy.GWW51V*) to 19551 bp (*Arahy.1E9R5B*), the CDS length ranged from 225 (*Arahy.GWW51V*) to 4104 bp (*Arahy.1E9R5B*), and the number of amino acid residues ranged from 74 to 1367. In addition, the GC content varied from 18.43% to 53.95% in *A. duranensis*, 32.03% to 47.88% in *A. ipaensis*, and 32.58% to 46.21% in *A. hypogaea* (Tables 1 and 2).

The characteristics of the RWP-RK proteins, including isoelectric point and molecular weight, were also analyzed. The isoelectric points ranged from 5.12 (Araip.KR88K) to 9.51 (Araip.80XBW) in *A. ipaensis*, 5.09 (Aradu.BBG0S) to 10.6 (Aradu.I1BME) in *A. duranensis*, and 5.03 (Arahy.DJ079B) to 10.29 (Arahy.GWW51V) in *A. hypogaea*. The molecular weights varied from 11464.91 (Araip.KR88K) to 109253.43 (Araip.R44NW) in *A. ipaensis*, 7137.65 (Aradu.I1BME) to 156101.58 (Aradu.G4SB3) in *A. duranensis*, and 8539.84 (Arahy.GWW51V) to 152021.99 (Arahy.1E9R5B) in *A. hypogaea* (Tables 1 and 2). Among these genes, 10 out of 16, 14 out of 19, and 22 out of 32 genes were predicted to be on the positive strand in *A. duranensis*, *A. ipaensis*, and *A. hypogaea*, respectively (Tables 1 and 2).

### 3.2. Chromosomal Location Analysis of *RWP-RK* Genes

**Figure 1:**
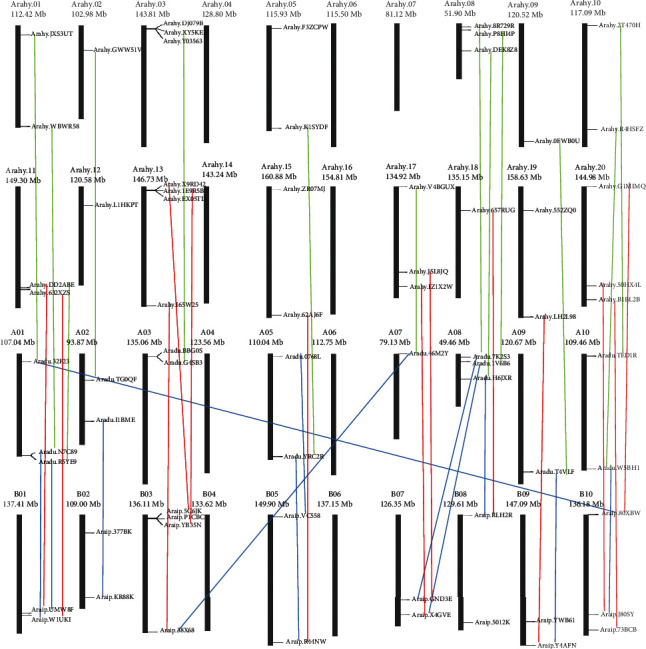
Chromosomal distribution of the *RWP-RK* genes in *A. duranensis* (AA genome), *A. ipaensis* (BB genome), and *A. hypogaea* (AABB genome). Orthologous gene pairs identified among the *A. duranensis*, *A. ipaensis*, and *A. hypogaea* genomes were connected by different colored lines.

To investigate the chromosomal locations of the peanut *RWP-RK* genes, we mapped them to their chromosomes in the wild and cultivated peanut genomes. The *RWP-RK* genes were unevenly distributed among chromosomes (Figure 1, Tables 1 and 2). In both wild peanut species, eight of the ten chromosomes contained the *RWP-RK* genes. Chromosomes 4 and 6 contained none (Figure 1, Tables 1 and 2). Chromosomes 1 and 8 contained the largest number of the *RWP-RK* genes in *A. duranensis*, with 3 members on each, whereas chromosome 3 contained the largest number of the *RWP-RK* genes in *A. ipaensis*, with 4 members. In *A. hypogaea*, 15 of the 20 chromosomes contained the *RWP-RK* genes. Chromosomes 4, 6, 7, 14, and 16 contained no *RWP-RK* genes, and chromosome 13 contained the largest number (4 genes), followed by chromosomes 3, 8, 17, and 20 (3 genes each) (Figure 1, Tables 1 and 2). Because the cultivated peanut AA and BB subgenomes are thought to derive from *A. duranensis* and *A. ipaensis*, respectively [25–29], the numbers of the *RWP-RK* genes in the wild species' chromosomes and the corresponding cultivated peanut chromosomes were analyzed. Six and seven of the ten chromosomes in *A. duranensis* and *A. ipaensis*, respectively, had the same *RWP-RK* gene numbers as their corresponding chromosomes in cultivated peanut. Chromosomes 1, 2, 3, and 7 in *A. duranensis* and chromosomes 2, 7, and 8 in *A. ipaensis* had numbers of the *RWP-RK* genes that differed from those of their corresponding chromosomes in cultivated peanut (Figure 1).

### 3.3. Classification and Phylogenetic Analysis of the *RWP-RK* Genes

**Figure 2:**
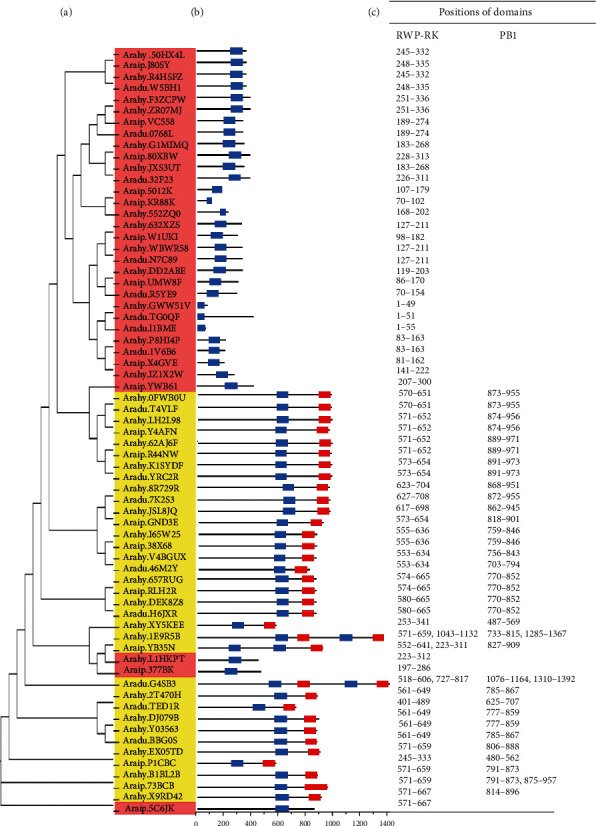
Phylogenetic and conserved domain analysis of the peanut RWP-RK proteins. (a) Phylogenetic analysis of the RWP-RK proteins from *A. duranensis*, *A. ipaensis*, and *A. hypogaea*. The pink and yellow highlights denote RKD and NLP proteins, respectively. (b) The positions of the conserved RWP and PB1 domains. The blue and red boxes indicate the RWP-RK and PB1 domains, respectively. (c) The position of each domain listed in (b).

The plant *RWP-RK* genes are mainly classified into two groups: *RKD* and *NLP* [4]. To investigate the evolutionary relationships among the peanut *RWP-RK* genes and to classify them into different subgroups, RWP-RK amino acid sequences from the wild and cultivated peanut genomes were used to construct a phylogenetic tree and analyze the conserved protein domains. The *A. duranensis*, *A. ipaensis*, and *A. hypogaea* genomes contained 8, 11, and 14 *RKD* members and 8, 8, and 18 *NLP* members, respectively (Tables 1 and 2, Figure 2). Conserved domain analysis revealed that most of the RKD genes were clustered together in a phylogenetic tree, while several RDK members showed close relationships with the *NLP* genes, including *Arahy.L1HKPT*, *Araip.YWB61*, *Araip.377BK*, and *Araip.5C6JK* (Figure 2). All the *RKD* members contained a single RWP-RK domain, and most of the *NLP* members contained one RWP domain and one PB1 domain. However, several NLP proteins contained inconsistent numbers of conserved domains. For example, Arahy.1E9R5B and Aradu.G4SB3 contained two RWP-RK domains and two PB1 domains, Araip.YB35N contained two RWP-RK domains and one PB1 domain, and Araip.73BCB contained one RWP-RK domain and two PB1 domains (Figure 2).

**Figure 3:**
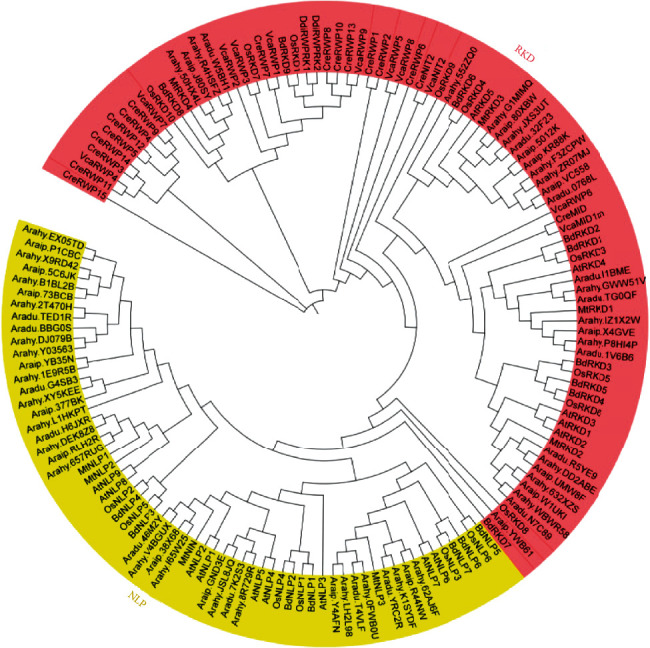
The evolutionary relationships among the RWP-RK proteins from peanuts. Amino acid sequences of the RWP-RK proteins from *A. duranensis*, *A. ipaensis*, *A. hypogaea*, *Arabidopsis*, *M. truncatula*, *B. dystachion*, *O. sativa*, *C. reinhardtii*, and *V. carteri* were used to construct a phylogenetic tree.

To obtain information from the well-studied *RWP-RK* genes of other species, we constructed a phylogenetic tree using *RWP-RK* amino acid sequences from the dicots *A. duranensis*, *A. ipaensis*, *A. hypogaea*, *Arabidopsis*, and *M. truncatula*; the monocots *Brachypodium distachyon* and *O. sativa*; and the algae *Chlamydomonas reinhardtii* and *Volvox carteri* described by Chardin et al. [4] (Figure 3). Among these genes, the *RKD* subgroup members *Aradu.1V6B6*, *Aradu.11BME*, *Aradu.TG0QF*, *Araip.X4GVE*, *Arahy.P8HI4P*, *Arahy.GWW51V*, and *Arahy.IZ1X2W* showed close relationships with *AtRKD4*, which controls cell differentiation during the female gametophyte development [19], suggesting that they may have critical functions in cell differentiation. In addition, the *NLP* members *Aradu.YRC2R* and *Aradu.T4VLF* in *A. duranensis*, *Araip.R44NW* and *Araip.Y4AFN* in *A. ipaensis*, and *Arahy.K1SYDF*, *Arahy.0FWB0U*, *Arahy.62AJ6F*, and *Arahy.LH2L98* in *A. hypogaea* showed close relationships with the nitrate response genes *AtNLP6* and *AtNLP7*, suggesting that they may be involved in the nitrate signal response in peanut [4, 15, 16]. *Aradu.46M2Y* in *A. duranensis*, *Araip.38X68* in *A. ipaensis*, and *Arahy.I65W25* and *Arahy.V4BGUX* in *A. hypogaea* showed close relationships with *MtNIN*, a well-studied gene involved in *M. truncatula* nodule formation [16], suggesting that these four genes may participate in nodule formation in peanut.

### 3.4. Orthologous Gene Pair Analysis

Many orthologous gene pairs have been identified among wild and cultivated peanuts [25–29], and we therefore investigated *RWP-RK* orthologs in the *A. duranensis*, *A. ipaensis*, and *A. hypogaea* genomes. A total of 38 orthologous gene pairs were found in the peanut genomes (Figure 1 and Figures S1–S3). The two wild peanut species shared twelve orthologous gene pairs, and each wild species shared thirteen orthologous gene pairs with A. hypogaea (Figure 1). Among the twelve orthologous pairs in the wild species, seven were found on syntenic loci of the *A. duranensis* and *A. ipaensis* chromosomes (Figure 1). By contrast, *Aradu.32F23*, *Aradu.46M2Y*, *Aradu.1V6B6*, and *Aradu.7K2S3* were located on different chromosomes from their *A. ipaensis* orthologs. Although *Aradu.H6JXR* and its *A. ipaensis* ortholog were both located on chromosome 8 in their respective genomes, they were located at different chromosomal positions (Figure 1). All orthologous gene pairs in *A. ipaensis* and *A. hypogaea* were found on the syntenic chromosomal loci, and 11 of the 13 gene pairs in *A. duranensis* and *A. hypogaea* were located on the syntenic chromosomal loci. These results suggest that the chromosomal rearrangement may have occurred in the diploid peanut genomes but not in that of cultivated peanut.

### 3.5. Gene Structures and Conserved Motifs of the *RWP-RK* Genes

**Figure 4:**
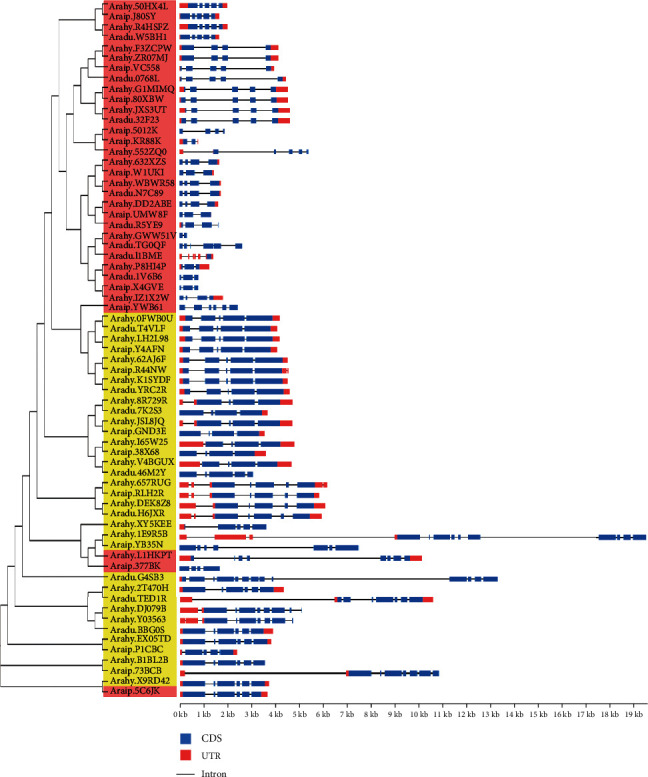
Exon-intron organizations of the *RWP-RK* genes.

**Figure 5:**
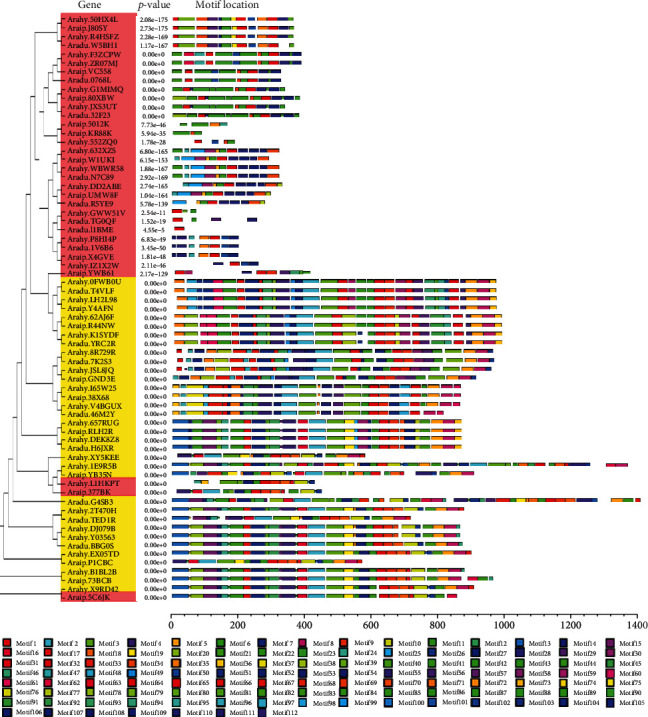
Conserved motif analysis of the RWP-RK proteins. Different motifs are indicated by colored boxes.

To investigate the structures of the *RWP-RK* genes, *RWP-RK* CDS and genomic sequences were downloaded from the peanut genome database and analyzed using the Gene Structure Display Server program [35]. Among the peanut *RWP-RK* members, 26 of the 33 *RKD* genes had predicted UTRs (except for *Araip.5012K*, *Araip.X4GVE*, *Araip.YWB61*, *Araip.377BK*, *Aradu.TG0QF*, *Aradu.1V6B6*, and *Arahy.GWW51V*), and 32 of the 34 *NLP* members had predicted UTRs (except for *Aradu.46M2Y* and *Araip.YB35N*) (Figure 4). For the *RKD* genes, exon numbers ranged from 1 to 8 and intron numbers ranged from 1 to 7, whereas for the *NLP* genes, exon numbers ranged from 4 to 13 and intron numbers ranged from 3 to 12. Moreover, 8 of the 12 orthologous pairs from *A. duranensis* and *A. ipaensis*, 8 of the 13 from *A. duranensis* and *A. hypogaea*, and 5 of the 13 from *A. ipaensis* and *A. hypogaea* had similar exon and intron numbers (Figures 1 and 4). MEME tool analysis identified 112 conserved motifs in the RWP-RK proteins (Figure 5 and Figure S4). Most of the NLP proteins had a greater number of conserved motifs than the RKD proteins, and many genes from the same clades had similar motif structures. For example, Arahy.50HX4L and Araip.J80SY had the same types and numbers of motifs, suggesting that these genes may be derived from a common ancestor.

### 3.6. Duplication Analysis of the *RWP-RK* Genes

**Figure 6:**
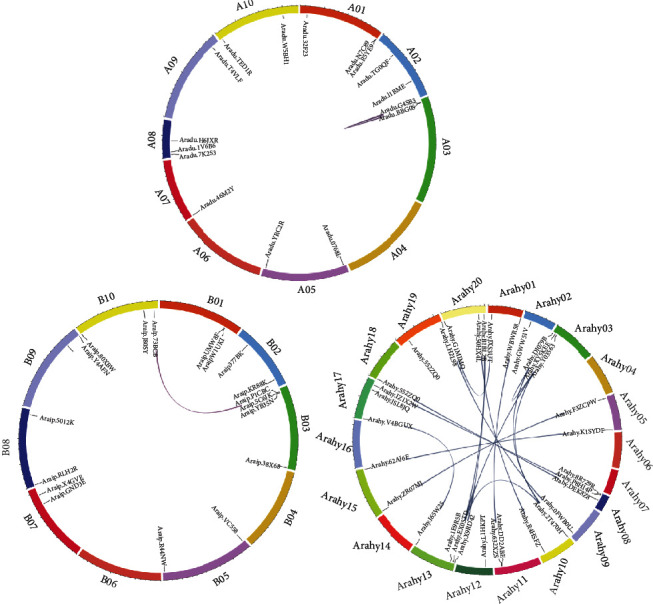
Duplication analysis of the *RWP-RK* genes in *A. duranensis* (AA genome), *A. ipaensis* (BB genome), and *A. hypogaea* (AABB genome).

The wild species *A. duranensis* and *A. ipaensis* are thought to have experienced one round of duplication, whereas the cultivated peanut *A. hypogaea* is thought to have experienced two rounds of duplication [25–29]. Gene duplication often occurs by polyploidization during the plant evolution [44], and therefore, we performed a homology analysis of the individual peanut genomes. One, one, and seventeen duplicated *RWP-RK* gene pairs were found within the *A. duranensis*, *A. ipaensis*, and *A. hypogaea* genomes, respectively. The duplicated genes *Aradu.BBG0S* and *Aradu.G4SB3* were located close to one another on chromosome 3 in *A. duranensis*, whereas *Araip.5C6JK* and *Araip.73BCB* were located on different chromosomes in *A. ipaensis* (Figure 6), highlighting the diversity of the two wild peanut genomes. In *A. hypogaea*, only eight *RWP-RK* genes had no duplicates, including four RKD genes (*Arahy.GWW51V*, *Arahy.DD2ABE*, *Arahy.L1HKPT*, and *Arahy.552ZQ0*) and four NLP genes (*Arahy.XY5KEE*, *Arahy.8R729R*, *Arahy.1E9R5B*, and *Arahy.JSL8JQ*). Among the duplicated genes in *A. hypogaea*, fifteen gene pairs were interchromosomal duplications, two duplications were located on the same chromosomes, and no tandem duplications were found. The *NLP* members formed eleven duplicated gene pairs: *Arahy.LH2L98/Arahy.0FWB0U*, *Arahy.Y03563/Arahy.2T470H*, *Arahy.DJ079B/Arahy.2T470H*, *Arahy.EX05TD/Arahy.2T470H*, *Arahy.DEK8Z8/Arahy.657RUG*, *Arahy.EX05TD/Arahy.B1BL2B*, *Arahy.X9RD42/Arahy.B1BL2B*, *Arahy.Y03563/Arahy.DJ079B*, *Arahy.X9RD42/Arahy.EX05TD*, *Arahy.Y03563/Arahy.EX05TD*, and *Arahy.V4BGUX/Arahy.I65W25*. The *RKD* members formed six duplicated gene pairs: *Arahy.R4HSFZ/Arahy.50HX4L*, *Arahy.WBWR58/Arahy.632XZS*, *Arahy.K1SYDF/Arahy.62AJ6F*, *Arahy.ZR07MJ/Arahy.F3ZCPW*, *Arahy.JXS3UT/Arahy.G1MIMQ*, and *Arahy.P8HI4P/Arahy.IZ1X2W* (Tables 1 and 2, Figure 6).

### 3.7. Analysis of *cis*-Acting Elements in Peanut *RWP-RK* Promoter Regions

**Figure 7:**
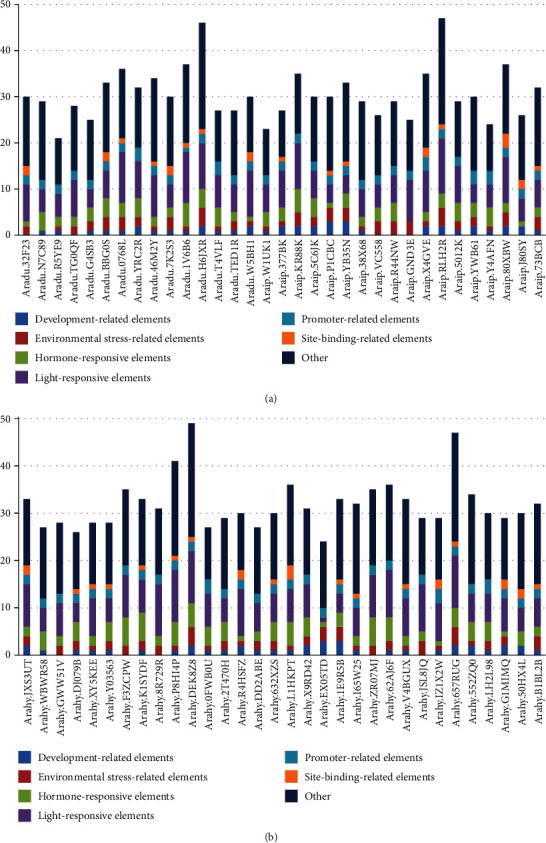
The number and type of *cis*-acting elements in each wild and cultivated peanut *RWP-RK* promoter region. (a) The number and type of *cis*-acting elements in each wild peanut *RWP-RK* promoter region. (b) The number and type of *cis*-acting elements in each cultivated peanut *RWP-RK* promoter region.

**Figure 8:**
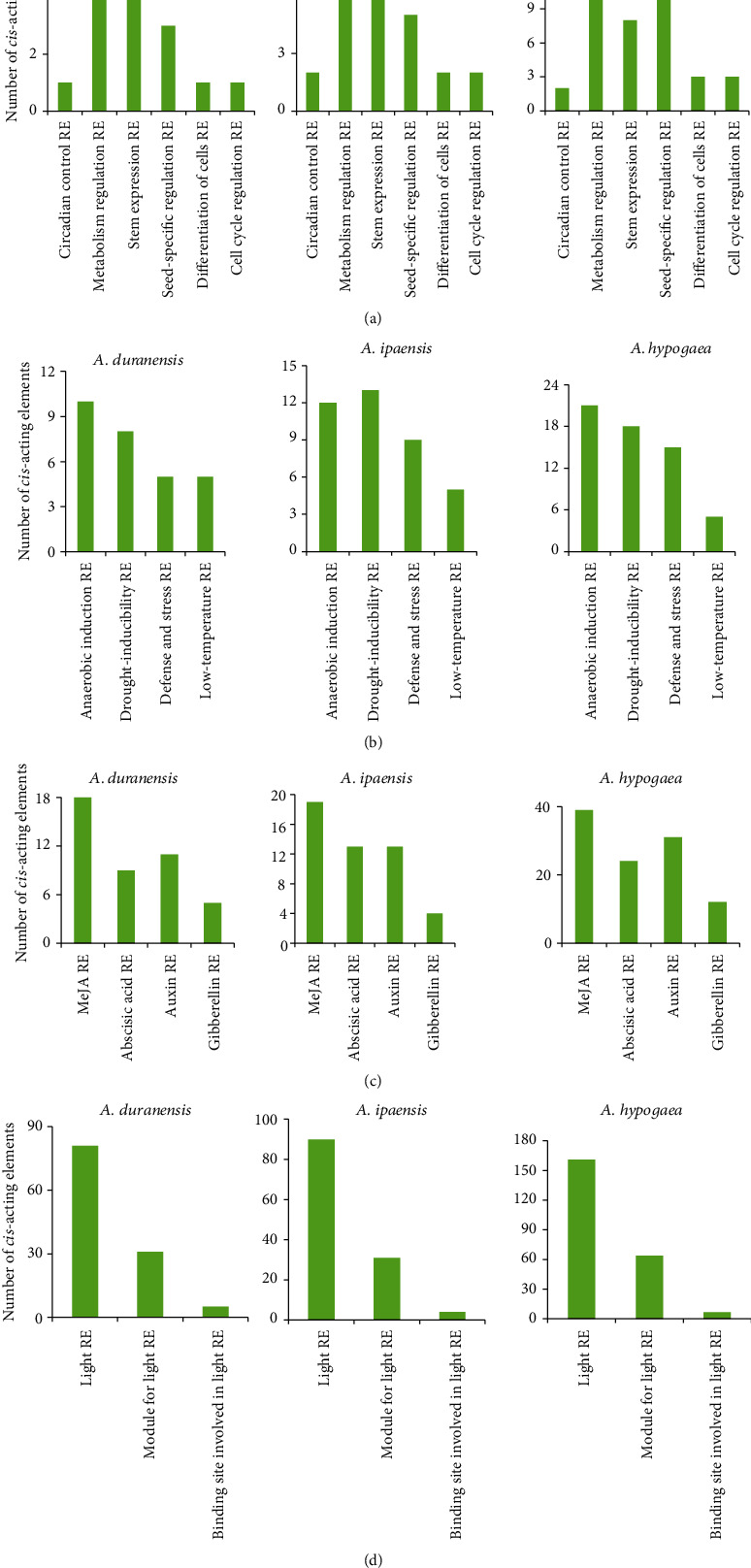
Analysis of *cis*-acting elements related to development, environmental stress, hormones, and light response in *RWP-RK* promoter regions of *A. duranensis*, *A. ipaensis*, and *A. hypogaea*. (a) Development-related *cis*-acting elements. (b) Environmental stress-related *cis*-acting elements. (c) Hormone-responsive *cis*-acting elements. (d) Light-responsive *cis*-acting elements.

To investigate the potential expression responses of the *RWP-RK* genes, we identified *cis*-acting elements in their promoter regions. *Aradu.I1BME* and *Araip.UMW8F* were discarded due to lack of promoter information. A total of 98 kinds of *cis*-acting elements were found across all peanut *RWP-RK* gene promoters (87 in *A. duranensis*, 89 in *A. ipaensis*, and 92 in *A. hypogaea*), 60 of which had predicted putative functions, including nine development-related elements (six in *A. duranensis*, nine in *A. ipaensis*, and eight in *A. hypogaea*), five environmental stress-related elements (four in *A. duranensis*, five in *A. ipaensis*, and four in *A. hypogaea*), ten hormone-responsive elements (eight in *A. duranensis*, eight in *A. ipaensis*, and ten in *A. hypogaea*), twenty-six light-responsive elements (twenty-five in *A. duranensis*, twenty-four in *A. ipaensis*, and twenty-five in *A. hypogaea*), four promoter-related elements (four in *A. duranensis*, four in *A. ipaensis*, and three in *A. hypogaea*), and six site-binding-related elements (five in *A. duranensis*, five in *A. ipaensis*, and six in *A. hypogaea*) (Figure 7, Tables S2-S5). Moreover, the numbers and types of *cis*-acting elements differed among the *RWP-RK* gene promoters, underscoring the functional diversity of these genes (Figure 7). The number of occurrences of each binding site differed between *A. duranensis* and *A. ipaensis* (Figure 8), and the number of occurrences of each binding site in *A. hypogaea* was close to the sum of its occurrences in *A. duranensis* and *A. ipaensis*. For example, *A. hypogaea* contained 39 methyl jasmonate (MeJA) response elements, and the sum of MeJA response elements in *A. duranensis* and *A. ipaensis* was 37 (18 in *A. duranensis* and 19 in *A. ipaensis*) (Figure 8). All *RWP-RK* promoters contained at least one light-responsive element, ranging from 4 to 11 in *A. duranensis*, 4 to 12 in *A. ipaensis*, and 1 to 11 in *A. hypogaea* (Figure 7, Table S2). Moreover, light-responsive elements were the most abundant element in each *RWP-RK* promoter, with the exception of *Arahy.EX05TD*. Sixty-one of the sixty-five *RWP-RK* genes contained the light-responsive element Box 4, suggesting that these genes function in light signaling pathways. The exceptions included *Aradu.7K2S3*, *Araip.P1CBC*, *Araip.377BK*, and *Arahy.EX05TD*. In addition, all of the *RWP-RK* promoters contained the promoter-related elements TATA-box and CAAT-box, which are responsible for the promoter function (Table S2).

### 3.8. Transcription Patterns of the Peanut *RWP-RK* Genes

**Figure 9:**
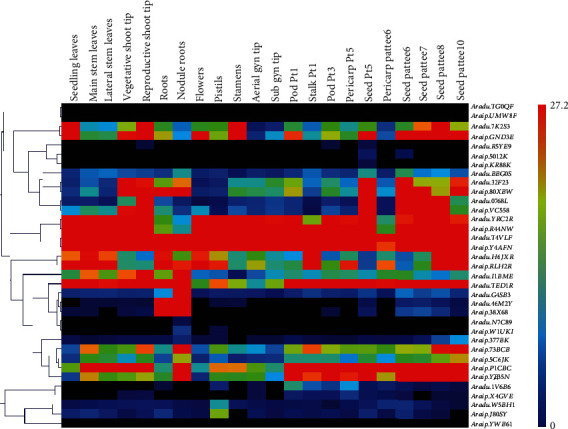
Expression profiles of the *RWP-RK* genes in 22 different tissues from two wild peanut species.

**Figure 10:**
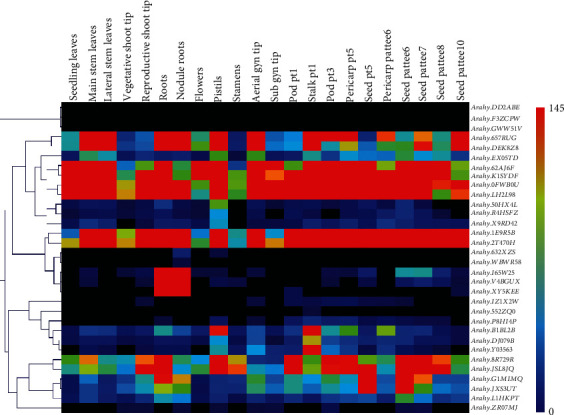
Expression profiles of the *RWP-RK* genes in 22 different tissues from cultivated peanut.

To investigate the transcription patterns of the peanut *RWP-RK* genes, heatmaps were constructed using RNA-seq datasets downloaded from the peanut database [41]. First, we used qRT-PCR analysis to verify the expression levels of several randomly selected *RWP-RK* genes in several tissues of wild and cultivated peanut. We found that their relative expression levels were similar to those in the published RNA-seq datasets (Figure S5). The *RWP-RK* genes were expressed at different levels in different tissues (Figures 9 and 10). Several genes were expressed at high levels in most of the tissues tested, including *A. duranensis* genes *Aradu.YRC2R* and *Aradu.T4VLF*, *A. ipaensis* genes *Araip.R44NW* and *Araip.Y4AFN*, and *A. hypogaea* genes *Arahy.62AJ6F*, *Arahy.K1SYDF*, *Arahy.0FWB0U*, *Arahy.LH2L98*, *Arahy.1E9R5B*, and *Arahy.2T470H* (Figures 9 and 10). By contrast, several genes showed extremely low expression levels in all tissues tested; these included *Aradu.TG0QF*, *Araip.UMW8F*, *Araip.YWB61*, *Arahy.DD2ABE*, *Arahy.F3ZCPW*, and *Arahy.GWW51V* (Figures 9 and 10). The *MtNIN* homologs *Aradu.46M2Y*, *Araip.38X68*, *Arahy.I65W25*, and *Arahy.V4BGUX* (Figure 3) were expressed at high levels in roots and nodule roots but at low levels elsewhere (Figures 9 and 10), suggesting that they may function in the nodule formation in peanut. In addition, most orthologous gene pairs showed similar expression levels in the two wild peanut species across many tissues (Figures 1 and 8). For example, the orthologs *Aradu.T4VLF* and *Araip.Y4AFN* showed similar expression levels in most tissues, with the exception of pericarp pattee 6. However, most orthologous gene pairs from *A. ipaensis* and *A. hypogaea* showed different expression levels, and similar results were found for *A. duranensis* and *A. hypogaea* orthologs (Figure 1 and Figure S6), highlighting the differences in gene expression between wild and cultivated peanut.

## 4. Discussion

The identification and characterization of the RWP-RK proteins have increased our understanding of nitrogen response and gametophyte development in many plant species [4, 15, 19]. Peanut is a globally important legume crop, and the characterization and comparative analysis of the RWP-RK proteins from wild and cultivated peanuts will increase our understanding of nitrate response and gametophyte development regulation in these species. In the current study, we identified and characterized 67 RWP-RK proteins from the wild species *A. duranensis* and *A. ipaensis* and from the cultivated peanut, *A. hypogaea*.

The *A. duranensis*, *A. ipaensis*, and *A. hypogaea* genomes contain 16, 19, and 32 RWP-RK proteins, respectively (Tables 1 and 2). The genome size of *A. hypogaea* is close to the sum of the *A. duranensis* and *A. ipaensis* genome sizes [25, 26], and the number of RWP-RK proteins in *A. hypogaea* is also close to the sum of those in *A. duranensis* and *A. ipaensis* (Tables 1 and 2). Although the two wild peanuts are diploid species, their numbers of RWP-RK proteins are different. The genome sizes of *A. duranensis*, *A. ipaensis*, and *A. hypogaea* are 1.25 Gb, 1.56 Gb, and 2.7 Gb, respectively [25–29], indicating that the number of RWP-RK proteins has changed during evolution and is directly related to the genome size in peanuts. By contrast, the *Arabidopsis*, rice, *B. distachyon*, and wheat genomes contain 14, 15, 18, and 37 RWP-RK proteins [4, 10], respectively, and their genome sizes are 125 Mb [45], 466 Mb [46], 260 Mb [47], and 17 Gb [48], respectively, suggesting that the number of RWP-RK proteins has no direct relationship with the genome size in other plant species. Approximately half of the RWP-RK proteins are classified as NLP members in vascular plants [4], including wild and cultivated peanuts. For example, the *Arabidopsis*, rice, *A. duranensis*, *A. ipaensis*, and *A. hypogaea* genomes contained nine (64% of all the RWP-RK proteins), six (40%), eight (50%), eight (42.11%), and 18 (56.25%) *NLP* members, respectively. The two diploid peanuts have the same number of NLP proteins, suggesting that the evolution of the *NLP* genes is conserved in the wild peanut species. However, the number of NLP proteins is expanded in *A. hypogaea* and is two greater than the sum of the *A. duranensis* and *A. ipaensis* NLP proteins. By contrast, the number of the RKD proteins in *A. hypogaea* is five less than the sum of the *A. duranensis* and *A. ipaensis* RKD proteins (Tables 1 and 2). These results indicate that the wild species have diverse numbers and types of the RWP-RK proteins and that the numbers and types of the RWP-RK proteins have changed during the evolution in the cultivated peanut.

*A. hypogaea* chromosomes 1 to 10 are thought to be derived from *A. duranensis*, and chromosomes 11 to 20 are thought to be derived from *A. ipaensis* [25–29]. Chromosomes 4 and 6 had no RWP-RK members in both *A. duranensis* and *A. ipaensis*, and thus, chromosomes 4, 6, 14, and 16 in *A. hypogaea* also had no *RWP-RK* genes (Figure 1). Chromosome 7 in *A. duranensis* contained one member, *Aradu.46M2Y*, but no *RWP-RK* gene was found on chromosome 7 in *A. hypogaea*. In addition, *A. hypogaea* chromosome 17 contained 3 *RWP-RK* members, whereas *A. ipaensis* chromosome 7 contained only 2 members. Moreover, the *Aradu.46M2Y* ortholog *Arahy.V4BGUX* was found on chromosome 17 in *A. hypogaea*, indicating that the chromosome rearrangement may have occurred between chromosomes 7 and 17 in *A. hypogaea*.

Wild and cultivated peanuts are thought to have experienced one and two rounds of duplication, respectively [25–29]. Many more orthologous gene pairs from *A. duranensis* and *A. ipaensis* (five out of 12) were located on different chromosomes than those from *A. ipaensis* and *A. hypogaea* (0 out of 13) or from *A. duranensis* and *A. hypogaea* (two out of 13) (Figure 1). In addition, among these orthologous gene pairs, eight of 12 from the two wild peanut species, eight of 13 from *A. duranensis* and *A. hypogaea*, and only five of 13 from *A. ipaensis* and *A. hypogaea* have similar exon and intron numbers (Figures 1 and 4). These results indicate that the chromosomal rearrangement may have occurred during the first round of duplication, and the gene structure alteration may have occurred during the second round of duplication, especially in the genes derived from *A. ipaensis*. Moreover, the orthologous gene pairs in the wild species showed similar expression levels in many tissues, whereas orthologous gene pairs from the wild species and cultivated peanut showed distinct expression patterns (Figures 9 and 10, Figure S6), indicating that the expression of the orthologs may have altered during the evolution of cultivated peanut.

*cis*-Acting elements in promoter regions are responsible for modulating the gene expression. We found different numbers and types of *cis*-acting elements in the *RWP-RK* promoters (Figure 7, Tables S2–S5), and these may be responsible for different expression levels of the *RWP-RK* genes in different tissues (Figures 9 and 10). However, some *RWP-RK* genes that contained many kinds of *cis*-acting elements in their promoters nonetheless showed extremely low expression levels in all the tested tissues (e.g., *Arahy.DD2ABE* and *Arahy.F3ZCPW*) (Figure 10). The gene expression is affected by many factors in addition to the presence of specific *cis*-acting elements. For example, epigenetic modifications, such as DNA methylation, have a substantial effect on the gene expression [49] and may be responsible for the low expression observed in these *RWP-RK* genes (Figure 10). Moreover, the expression of orthologous genes from the two wild peanuts was similar in many tissues, whereas orthologous genes from wild and cultivated peanut showed different expression levels (Figures 9 and 10). The wild peanut experienced one round of whole genome duplication, whereas cultivated peanut experienced two rounds of whole genome duplication, and this may explain their different tissue-specific gene expression patterns. In addition, *Aradu.YRC2R* and *Aradu.T4VLF* from *A. duranensis*, *Araip.R44NW* and *Araip.Y4AFN* from *A. ipaensis*, and *Arahy.K1SYDF*, *Arahy.0FWB0U*, *Arahy.62AJ6F*, and *Arahy.LH2L98* from *A. hypogaea* showed close relationships with the nitrate response genes *AtNLP6* and *AtNLP7* (Figure 3). However, the expression level of these genes under N-limited conditions was similar to that under normal conditions, suggesting that their gene expression was not regulated by N (Figure S7).

Gene duplication occurs on various scales during evolution, including segmental, tandem, and whole genome duplications [50]. The cultivated peanut is descended from the hybridization and polyploidization of two wild diploids, which resulted in whole genome duplication and produced many duplicated gene pairs [25–29]. Thus, up to 17 duplicated *RWP-RK* gene pairs were found in the genome of cultivated peanut. In contrast, only one duplication event, which appears to be a segmental duplication, was found in each of the two wild species (Figure 6). Duplicated genes can be lost, pseudogenized, or become novel genes during evolution [30, 51]. The two *RWP-RK* genes from each duplication event belong to the same subfamily in *A. duranensis* and *A. hypogaea*, and these duplicated gene pairs may retain many similar functions because of their shared origin. In contrast, *Araip.73BCB* belonged to the *NLP* subgroup, but its duplicate gene *Araip.5C6JK* was an *RKD* member. *Araip.73BCB* contained two PB1 domains whereas *Araip.5C6JK* had no PB1 domain, and these two genes showed different expression patterns in many tissues (Figure 9), indicating their functional differentiation. In addition, the amino acid numbers of most RKD proteins other than Araip.5C6JK were less than those of NLP proteins (Tables 1 and 2, Figure 2). These results suggest that the PB1 domain of *Araip.73BCB* may have been lost and the remainder of the gene retained to give rise to a new gene, *Araip.5C6JK*.

## 5. Conclusions

In summary, many characteristics of the RWP-RK proteins were analyzed in wild and cultivated peanuts, including chromosomal locations, gene structures, orthologous gene pairs, conserved motifs, duplications, phylogenetic relationships, *cis*-acting elements, and transcription patterns. Although the two diploid peanuts *A. duranensis* and *A. ipaensis* had some common features, the *RWP-RK* genes in these two wild species also showed some degree of diversity. In addition, while the *RWP-RK* genes of cultivated peanut retained some characteristics of those from wild peanuts, they also changed during evolution compared with those of their two diploid ancestors.

## Figures and Tables

**Table 1:** The *RWP-RK* genes identified from wild peanuts *A. duranensis* and *A. ipaensis*.

Gene ID	Genomic length (bp)	CDS (bp)	Size/AA	pI	MW	Chr	Strand	GC (%)	Groups
*Aradu.32F23*	4586	1140	379	9.6	43697.19	A01	+	38.51%	RKD
*Aradu.N7C89*	1709	978	325	5.17	37811.5	A01	+	37.61%	RKD
*Aradu.R5YE9*	1614	861	286	7.56	33081.56	A01	−	38.61%	RKD
*Aradu.TG0QF*	2597	1227	408	10.42	44980.31	A02	−	53.95%	RKD
*Aradu.I1BME*	1395	180	59	10.6	7137.65	A02	+	40.04%	RKD
*Aradu.G4SB3*	13327	4212	1403	5.78	156101.58	A03	+	42.69%	NLP
*Aradu.BBG0S*	3904	2616	871	5.09	96462.81	A03	−	40.70%	NLP
*Aradu.0768L*	4428	990	329	6.24	38019.69	A05	+	18.43%	RKD
*Aradu.YRC2R*	4577	2961	986	5.49	109465.76	A05	+	40.83%	NLP
*Aradu.46M2Y*	3055	2442	813	5.89	89573.68	A07	−	53.40%	NLP
*Aradu.7K2S3*	3661	2907	968	5.96	108152.61	A08	+	41.24%	NLP
*Aradu.1V6B6*	779	609	202	5.27	23804.12	A08	+	40.91%	RKD
*Aradu.H6JXR*	5952	2613	870	6.51	96812.14	A08	−	39.76%	NLP
*Aradu.T4VLF*	4064	2904	967	5.59	107545.98	A09	−	42.24%	NLP
*Aradu.TED1R*	10616	2157	718	5.34	79574.8	A10	+	39.19%	NLP
*Aradu.W5BH1*	1637	1092	363	5.49	40985.92	A10	+	40.08%	RKD
*Araip.UMW8F*	1318	891	296	6.16	33981.41	B01	+	35.44%	RKD
*Araip.W1UKI*	1428	882	293	5.97	33835.29	B01	−	32.78%	RKD
*Araip.377BK*	1668	1380	459	8.72	50670.58	B02	+	38.49%	RKD
*Araip.KR88K*	771	309	102	5.12	11464.91	B02	+	37.09%	RKD
*Araip.5C6JK*	3681	2565	854	5.61	94141.19	B03	+	39.09%	RKD
*Araip.P1CBC*	2396	1722	573	6.56	63372.57	B03	+	39.35%	NLP
*Araip.YB35N*	7492	2739	912	6.41	100976.79	B03	+	32.54%	NLP
*Araip.38X68*	3584	2598	865	6.06	95607.18	B03	+	47.88%	NLP
*Araip.VC558*	3927	990	329	6.24	37987.65	B05	+	34.99%	RKD
*Araip.R44NW*	4540	2955	984	5.42	109253.43	B05	+	38.59%	NLP
*Araip.GND3E*	3543	2745	914	5.67	102372.56	B07	−	38.81%	NLP
*Araip.X4GVE*	772	600	199	5.28	23612.04	B07	+	39.50%	RKD
*Araip.RLH2R*	5851	2613	870	6.41	96849.27	B08	−	36.64%	NLP
*Araip.5012K*	1869	540	179	9.22	19935.95	B08	+	36.22%	RKD
*Araip.YWB61*	2408	1263	420	8.95	46784.46	B09	−	42.94%	RKD
*Araip.Y4AFN*	4061	2910	969	5.63	107839.27	B09	+	40.04%	NLP
*Araip.80XBW*	4523	1146	381	9.51	43893.44	B10	+	35.62%	RKD
*Araip.J80SY*	1652	1092	363	5.78	40913.96	B10	+	36.68%	RKD
*Araip.73BCB*	10861	2880	959	5.32	106753.86	B10	−	32.03%	NLP

Chr: chromosome; MW: molecular weight; pI: isoelectric point.

**Table 2:** The *RWP-RK* genes identified from cultivated peanut *A. hypogaea*.

Gene ID	Genomic length (bp)	CDS (bp)	Size/AA	pI	MW	Chromosome	Strand	GC (%)	Groups
*Arahy.JXS3UT*	4585	1011	336	9.47	38823.45	Arahy.01	−	38.48%	RKD
*Arahy.WBWR58*	1717	978	325	5.12	37869.53	Arahy.01	+	32.90%	RKD
*Arahy.GWW51V*	291	225	74	10.29	8539.84	Arahy.02	−	36.00%	RKD
*Arahy.DJ079B*	5106	2664	887	5.03	98664.42	Arahy.03	+	39.73%	NLP
*Arahy.XY5KEE*	3606	1743	580	8.29	64531.34	Arahy.03	+	32.58%	NLP
*Arahy.Y03563*	4733	2622	873	5.12	97058.43	Arahy.03	−	39.01%	NLP
*Arahy.F3ZCPW*	4111	1176	391	7.61	45229.04	Arahy.05	+	38.93%	RKD
*Arahy.K1SYDF*	4492	2961	986	5.48	109443.61	Arahy.05	+	41.48%	NLP
*Arahy.8R729R*	4694	2895	964	5.63	107484.54	Arahy.08	+	40.20%	NLP
*Arahy.P8HI4P*	1235	609	202	5.39	23873.23	Arahy.08	+	38.77%	RKD
*Arahy.DEK8Z8*	6111	2613	870	6.51	96789.10	Arahy.08	−	39.24%	NLP
*Arahy.0FWB0U*	4166	2904	967	5.59	107515.96	Arahy.09	−	42.15%	NLP
*Arahy.2T470H*	4338	2637	878	5.20	97311.82	Arahy.10	+	40.47%	NLP
*Arahy.R4HSFZ*	1985	1083	360	5.49	40503.32	Arahy.10	+	38.09%	RKD
*Arahy.DD2ABE*	1604	990	329	5.57	37937.79	Arahy.11	+	39.07%	RKD
*Arahy.632XZS*	1646	969	322	5.41	37396.10	Arahy.11	−	38.18%	RKD
*Arahy.L1HKPT*	10131	1320	439	9.11	49083.34	Arahy.12	+	38.01%	RKD
*Arahy.X9RD42*	3730	2724	907	5.29	100095.84	Arahy.13	+	39.08%	NLP
*Arahy.EX05TD*	3825	2700	899	5.11	99460.11	Arahy.13	+	41.63%	NLP
*Arahy.1E9R5B*	19551	4104	1367	5.68	152021.99	Arahy.13	+	40.09%	NLP
*Arahy.I65W25*	4809	2598	865	6.06	95607.18	Arahy.13	+	45.48%	NLP
*Arahy.ZR07MJ*	4111	1176	391	7.61	45229.04	Arahy.15	+	34.61%	RKD
*Arahy.62AJ6F*	4492	2955	984	5.42	109253.43	Arahy.15	+	41.38%	NLP
*Arahy.V4BGUX*	4661	2589	862	6.10	95387.07	Arahy.17	+	46.21%	NLP
*Arahy.JSL8JQ*	4687	2877	958	5.60	106770.63	Arahy.17	−	39.77%	NLP
*Arahy.IZ1X2W*	1795	813	270	7.06	31094.36	Arahy.17	+	40.64%	RKD
*Arahy.657RUG*	6373	2613	870	6.41	96849.27	Arahy.18	−	39.59%	NLP
*Arahy.552ZQ0*	5406	669	222	8.79	25083.55	Arahy.19	−	37.31%	RKD
*Arahy.LH2L98*	4163	2910	969	5.63	107839.27	Arahy.19	+	41.91%	NLP
*Arahy.G1MIMQ*	4509	1011	336	9.41	38823.49	Arahy.20	+	38.94%	RKD
*Arahy.50HX4L*	1977	1083	360	5.68	40521.48	Arahy.20	+	37.40%	RKD
*Arahy.B1BL2B*	3569	2628	875	5.46	97007.68	Arahy.20	−	41.71%	NLP

## Data Availability

The original data of the RWP-RK family genes are available from the peanut genome database (https://www.peanutbase.org/).

## Supplementary Materials

Supplementary 1 Figure S1: phylogenetic relationship analysis of the RWP-RK proteins from *A. duranensis* and *A. ipaensis*. Figure S2: phylogenetic relationship analysis of the RWP-RK proteins from *A. duranensis* and *A. hypogaea*. Figure S3: phylogenetic relationship analysis of the RWP-RK proteins from *A. ipaensis* and *A. hypogaea*. Figure S4: sequence logos of motifs in the peanut RWP-RK proteins. The “sites” and “width” indicate the number of RWP-RK proteins containing each motif and the amino acid number of each motif, respectively. Figure S5: expression patterns of several wild and cultivated peanut RWP-RK genes in five selected tissues. For genes in *A. ipaensis*, the expression level of *Araip.R44NW* in seedling leaves was set as 1, and the others were adjusted accordingly. For genes in *A. duranensis*, the expression level of *Aradu.T4VLF* in seedling leaves was set as 1, and the others were adjusted accordingly. For genes in *A. hypogaea*, the expression level of *Arahy.0FWB0U* in seedling leaves was set as 1, and the others were adjusted accordingly. Figure S6: expression profiles of the RWP-RK genes in 22 different tissues in wild and cultivated peanuts. Figure S7: the expressions of the selected genes under normal and N-free conditions. For N free treatment, 10-day-old peanut plants were grown in normal and N-free solution culture for 1 (1 d) and 3 days (3 d), respectively. Plants grown under normal conditions were sampled as the control. The whole plants were sampled. The expression level of each gene in the control was set as 1, and the others were adjusted accordingly.

Supplementary 2 Table S1: primers used in this study.

Supplementary 3 Table S2: function analysis of the *cis*-acting elements in peanut *RWP-RK* promoter regions. The classifications, names, and putative functions of related *cis*-acting elements are listed.

Supplementary 4 Table S3: function analysis of the *cis*-acting elements in *A. duranensis* RWP-RK promoter regions. The classifications, names, and putative functions of related *cis*-acting elements are listed.

Supplementary 5 Table S4: function analysis of the *cis*-acting elements in *A. ipaensis RWP-RK* promoter regions. The classifications, names, and putative functions of related *cis*-acting elements are listed.

Supplementary 6 Table S5: function analysis of the *cis*-acting elements in *A. hypogaea RWP-RK* promoter regions. The classifications, names, and putative functions of related *cis*-acting elements are listed.
