# Supplementary material for: Characterization and Comparative Analysis of RWP-RK Proteins from Arachis duranensis, Arachis ipaensis, and Arachis hypogaea
Source: Int J Genomics. 2020 Aug 27;2020:2568640. doi: 10.1155/2020/2568640 (PMC7474775; doi:10.1155/2020/2568640)
Supplement: Supplementary 1 — Figure S1: phylogenetic relationship analysis of the RWP-RK proteins from A. duranensis and A. ipaensis. Figure S2: phylogenetic relationship analysis of the RWP-RK proteins from A. duranensis and A. hypogaea. Figure S3: phylogenetic relationship analysis of the RWP-RK proteins from A. ipaensis and A. hypogaea. Figure S4: sequence logos of motifs in the peanut RWP-RK proteins. The “sites” and “width” indicate the number of RWP-RK proteins containing each motif and the amino acid number of each motif, respectively. Figure S5: expression patterns of several wild and cultivated peanut RWP-RK genes in five selected tissues. For genes in A. ipaensis, the expression level of Araip.R44NW in seedling leaves was set as 1, and the others were adjusted accordingly. For genes in A. duranensis, the expression level of Aradu.T4VLF in seedling leaves was set as 1, and the others were adjusted accordingly. For genes in A. hypogaea, the expression level of Arahy.0FWB0U in seedling leaves was set as 1, and the others were adjusted accordingly. Figure S6: expression profiles of the RWP-RK genes in 22 different tissues in wild and cultivated peanuts. Figure S7: the expressions of the selected genes under normal and N-free conditions. For N free treatment, 10-day-old peanut plants were grown in normal and N-free solution culture for 1 (1 d) and 3 days (3 d), respectively. Plants grown under normal conditions were sampled as the control. The whole plants were sampled. The expression level of each gene in the control was set as 1, and the others were adjusted accordingly. [file 2568640.f1.pptx]

## Slide 1
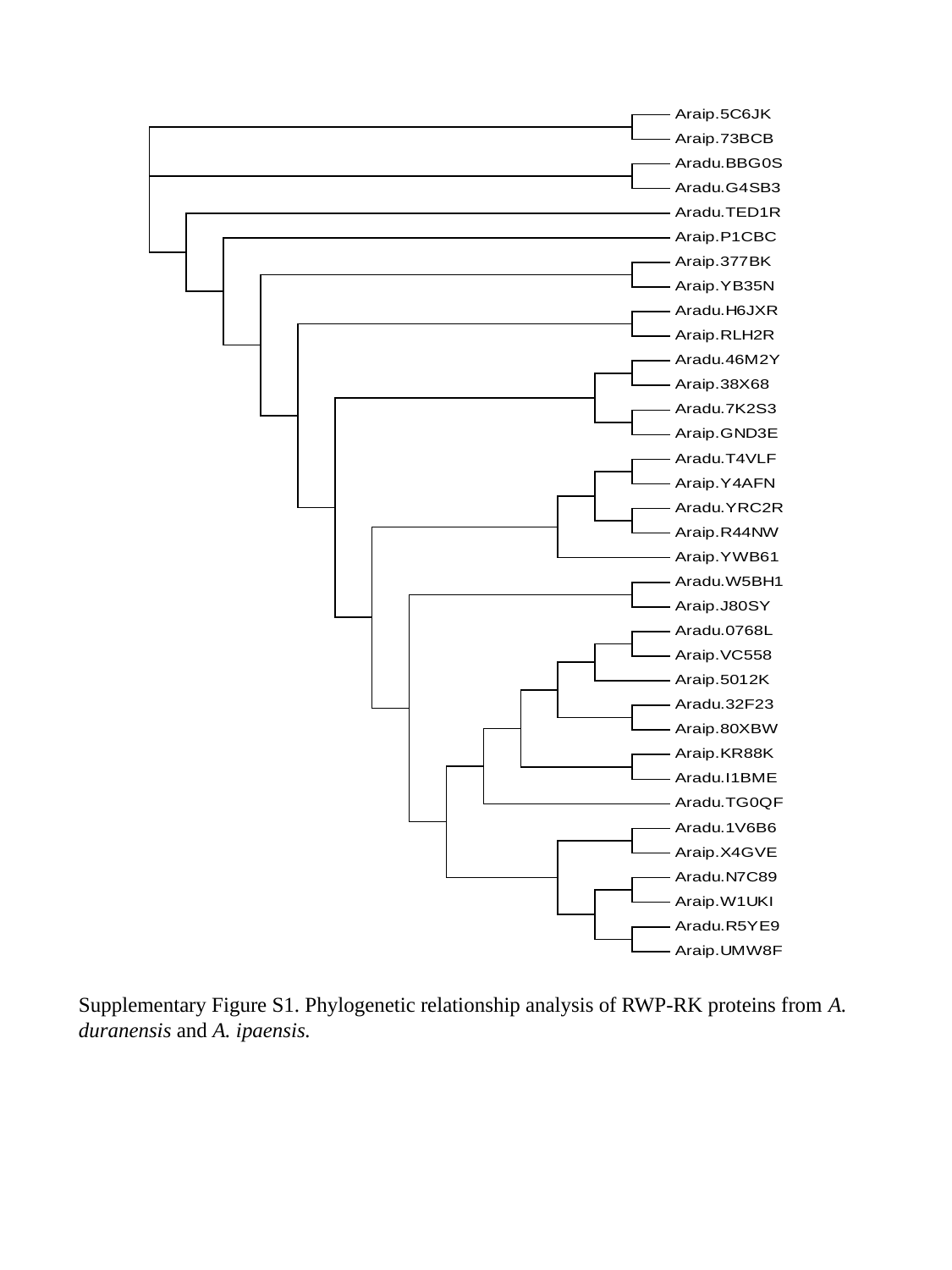

Supplementary Figure S1. Phylogenetic relationship analysis of RWP-RK proteins from A. duranensis and A. ipaensis.

## Slide 2
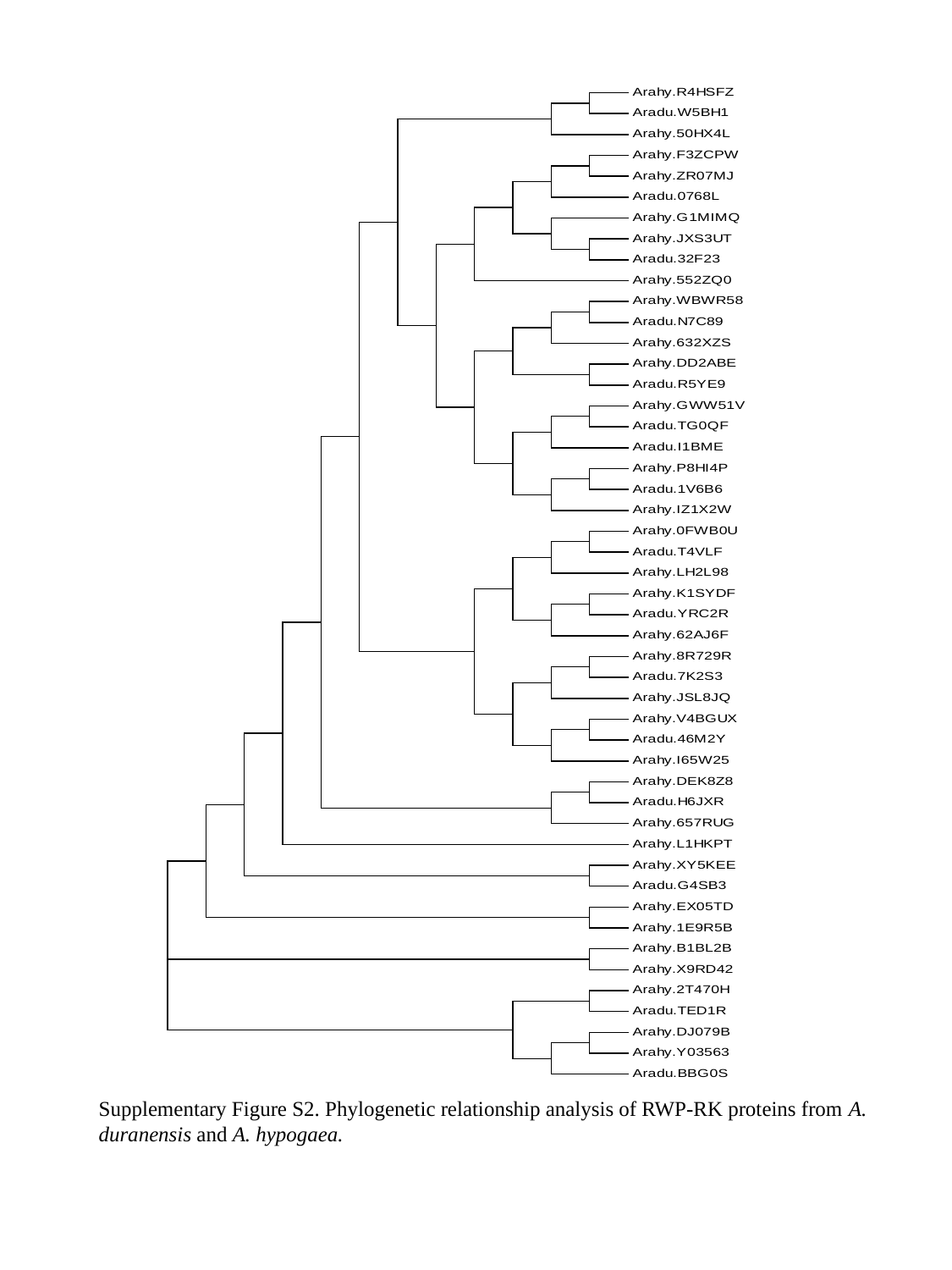

Supplementary Figure S2. Phylogenetic relationship analysis of RWP-RK proteins from A. duranensis and A. hypogaea.

## Slide 3
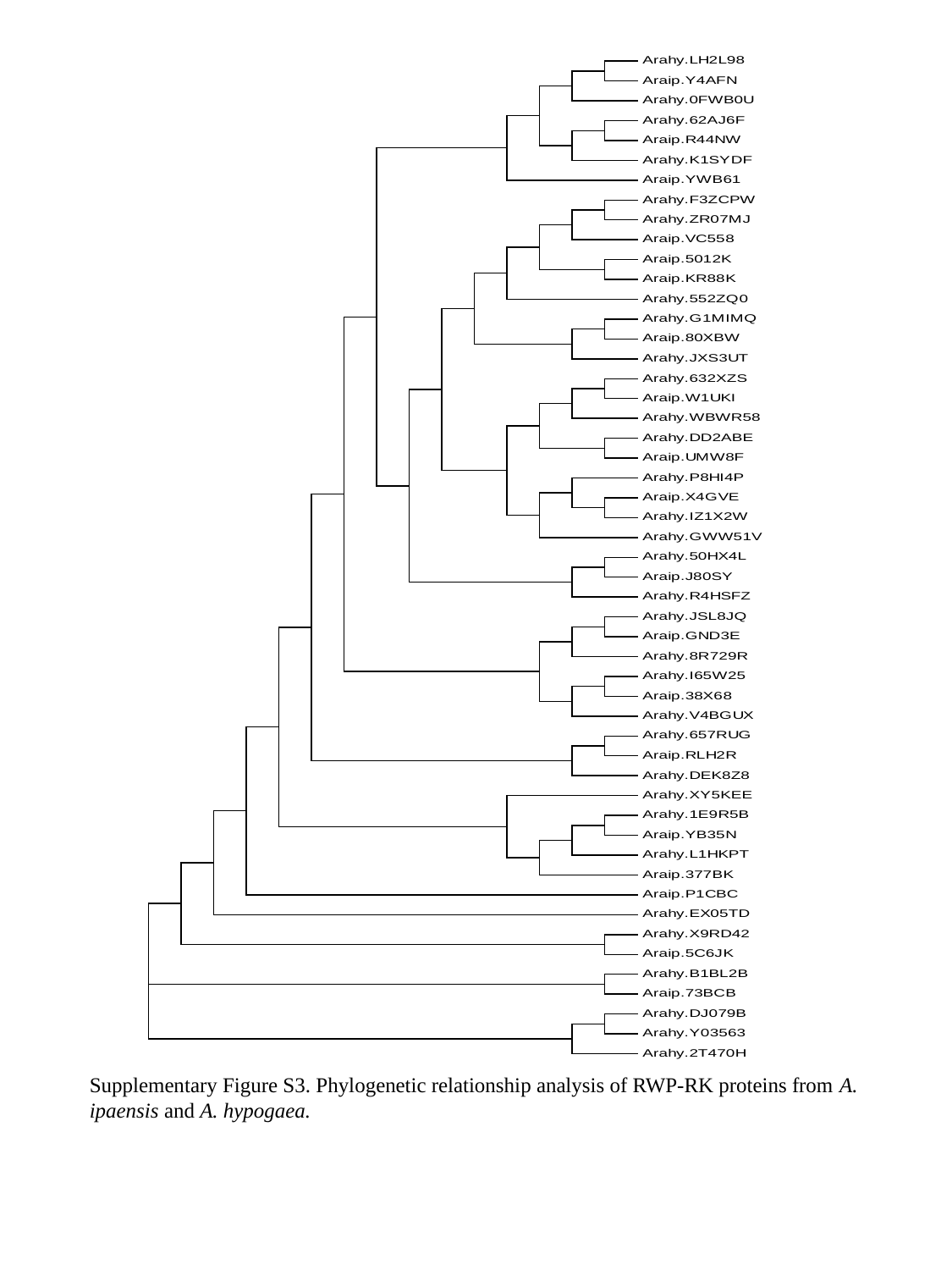

Supplementary Figure S3. Phylogenetic relationship analysis of RWP-RK proteins from A. ipaensis and A. hypogaea.

## Slide 4
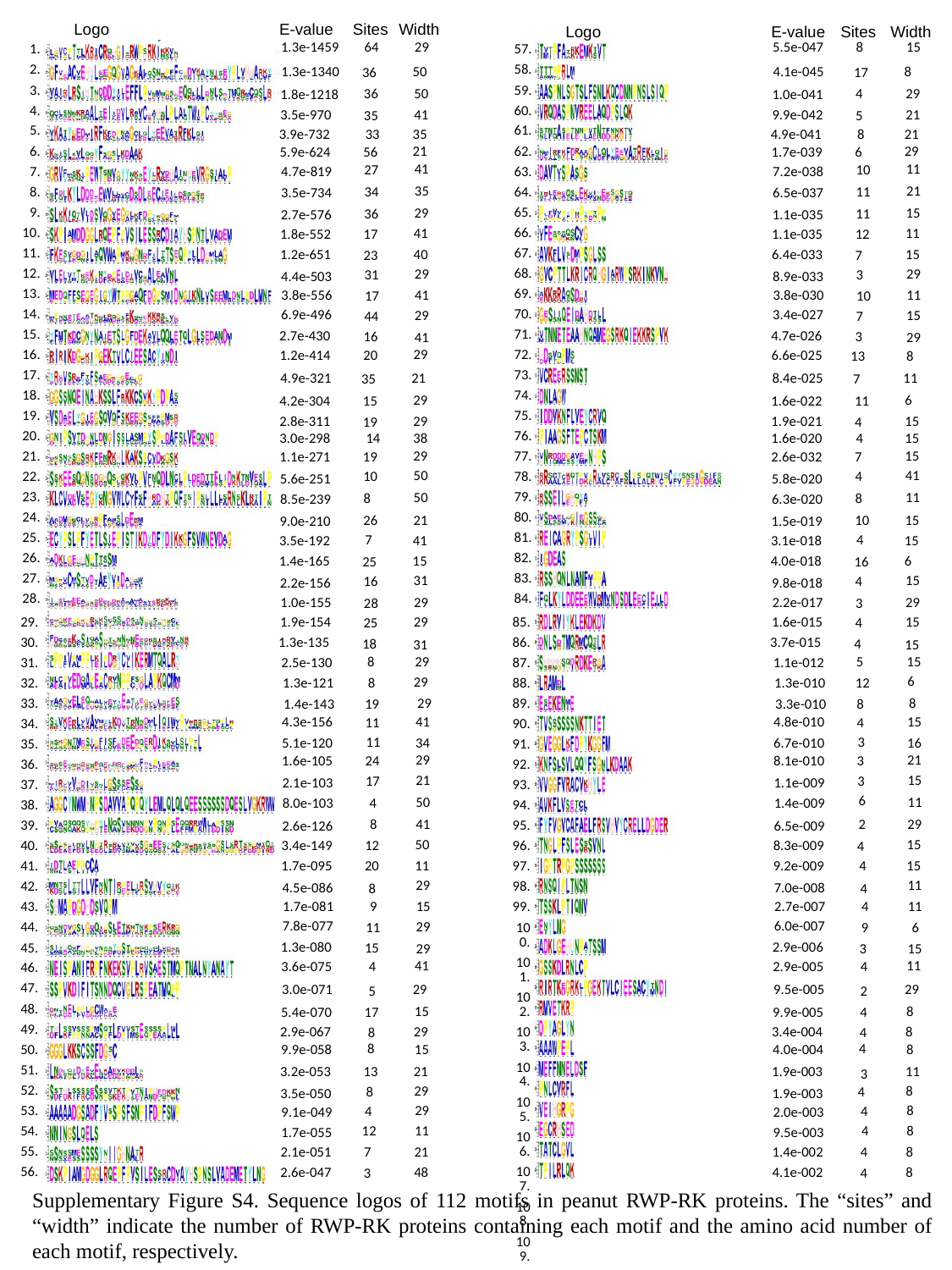

Logo
E-value
Sites
Width
29
1.3e-1459
64
50
1.3e-1340
36
50
36
1.8e-1218
41
3.5e-970
35
33
3.9e-732
35
21
5.9e-624
56
41
27
4.7e-819
35
34
3.5e-734
29
36
2.7e-576
41
1.8e-552
17
40
1.2e-651
23
29
31
4.4e-503
41
3.8e-556
17
6.9e-496
29
44
2.7e-430
16
41
29
1.2e-414
20
21
4.9e-321
35
29
15
4.2e-304
29
2.8e-311
19
14
3.0e-298
38
29
1.1e-271
19
50
10
5.6e-251
50
8
8.5e-239
21
26
9.0e-210
7
41
3.5e-192
15
1.4e-165
25
31
16
2.2e-156
29
1.0e-155
28
1.9e-154
29
25
1.3e-135
18
31
8
29
2.5e-130
29
1.3e-121
8
29
1.4e-143
19
41
4.3e-156
11
11
5.1e-120
34
29
1.6e-105
24
21
17
2.1e-103
50
8.0e-103
4
8
41
2.6e-126
50
3.4e-149
12
11
1.7e-095
20
29
4.5e-086
8
9
15
1.7e-081
7.8e-077
29
11
1.3e-080
15
29
41
3.6e-075
4
29
3.0e-071
5
15
17
5.4e-070
29
2.9e-067
8
8
9.9e-058
15
21
3.2e-053
13
29
8
3.5e-050
29
4
9.1e-049
12
11
1.7e-055
21
2.1e-051
7
48
2.6e-047
3
Logo
E-value
Sites
Width
15
5.5e-047
8
8
4.1e-045
17
29
4
1.0e-041
21
9.9e-042
5
8
4.9e-041
21
29
1.7e-039
6
11
10
7.2e-038
21
11
6.5e-037
15
11
1.1e-035
11
1.1e-035
12
15
6.4e-033
7
29
3
8.9e-033
11
3.8e-030
10
3.4e-027
15
7
4.7e-026
3
29
6.6e-025
8
13
11
8.4e-025
7
6
11
1.6e-022
15
1.9e-021
4
1.6e-020
4
15
15
2.6e-032
7
41
4
5.8e-020
11
8
6.3e-020
15
10
1.5e-019
4
15
3.1e-018
6
4.0e-018
16
15
4
9.8e-018
29
2.2e-017
3
1.6e-015
15
4
3.7e-015
4
15
5
15
1.1e-012
6
1.3e-010
12
8
3.3e-010
8
15
4.8e-010
4
3
6.7e-010
16
21
8.1e-010
3
15
3
1.1e-009
6
11
1.4e-009
2
29
6.5e-009
15
8.3e-009
4
15
9.2e-009
4
11
7.0e-008
4
4
11
2.7e-007
6.0e-007
9
6
2.9e-006
15
3
11
2.9e-005
4
29
9.5e-005
2
8
4
9.9e-005
8
3.4e-004
4
4
4.0e-004
8
11
1.9e-003
3
8
4
1.9e-003
8
4
2.0e-003
4
8
9.5e-003
8
1.4e-002
4
8
4.1e-002
4
| 1. |
| --- |
| 2. |
| 3. |
| 4. |
| 5. |
| 6. |
| 7. |
| 8. |
| 9. |
| 10. |
| 11. |
| 12. |
| 13. |
| 14. |
| 15. |
| 16. |
| 17. |
| 18. |
| 19. |
| 20. |
| 21. |
| 22. |
| 23. |
| 24. |
| 25. |
| 26. |
| 27. |
| 28. |
| 57. |
| --- |
| 58. |
| 59. |
| 60. |
| 61. |
| 62. |
| 63. |
| 64. |
| 65. |
| 66. |
| 67. |
| 68. |
| 69. |
| 70. |
| 71. |
| 72. |
| 73. |
| 74. |
| 75. |
| 76. |
| 77. |
| 78. |
| 79. |
| 80. |
| 81. |
| 82. |
| 83. |
| 84. |
| 29. |
| --- |
| 30. |
| 31. |
| 32. |
| 33. |
| 34. |
| 35. |
| 36. |
| 37. |
| 38. |
| 39. |
| 40. |
| 41. |
| 42. |
| 43. |
| 44. |
| 45. |
| 46. |
| 47. |
| 48. |
| 49. |
| 50. |
| 51. |
| 52. |
| 53. |
| 54. |
| 55. |
| 56. |
| 85. |
| --- |
| 86. |
| 87. |
| 88. |
| 89. |
| 90. |
| 91. |
| 92. |
| 93. |
| 94. |
| 95. |
| 96. |
| 97. |
| 98. |
| 99. |
| 100. |
| 101. |
| 102. |
| 103. |
| 104. |
| 105. |
| 106. |
| 107. |
| 108. |
| 109. |
| 110. |
| 111. |
| 112. |
Supplementary Figure S4. Sequence logos of 112 motifs in peanut RWP-RK proteins. The “sites” and “width” indicate the number of RWP-RK proteins containing each motif and the amino acid number of each motif, respectively.

## Slide 5
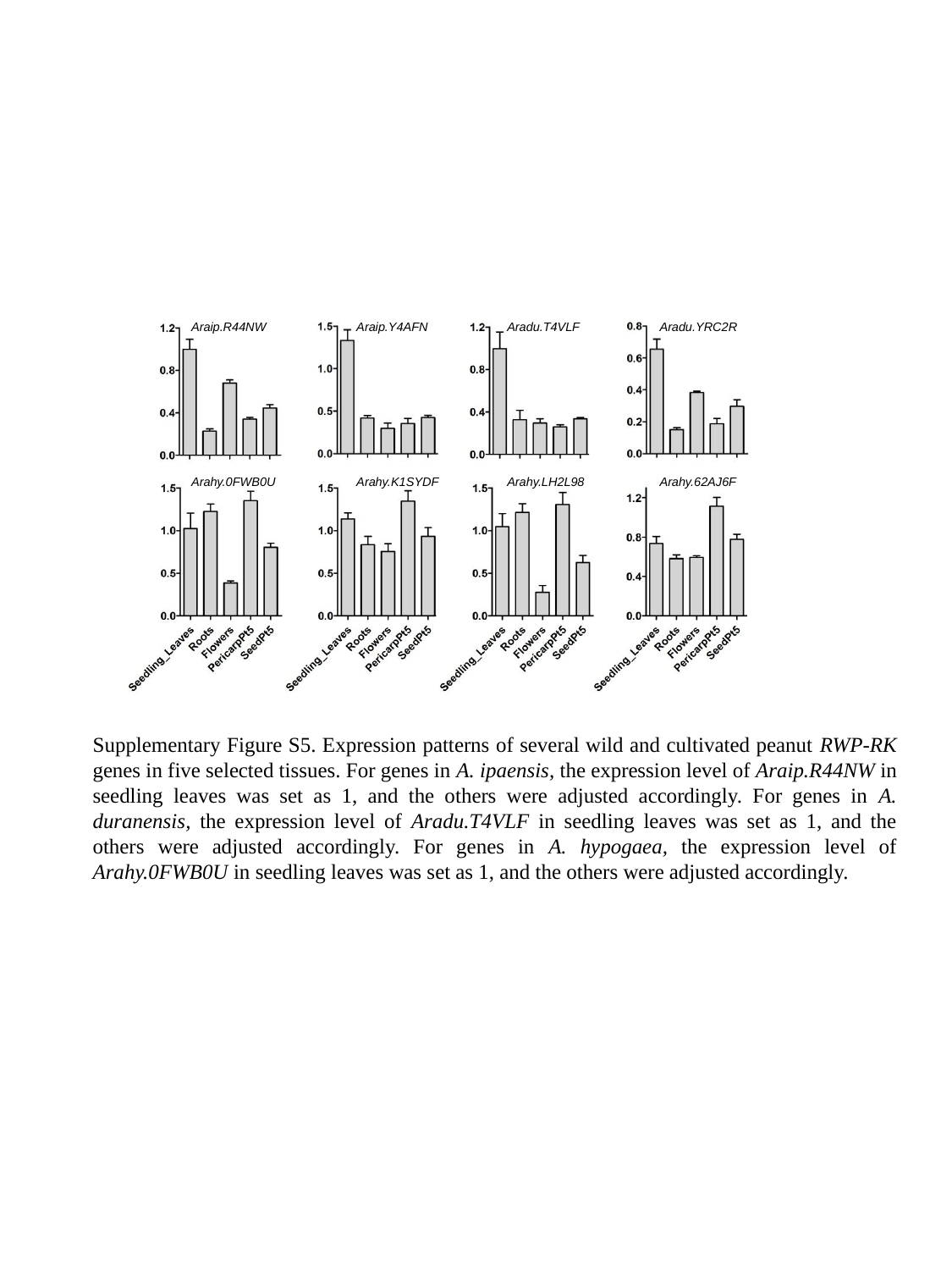

Araip.R44NW
Araip.Y4AFN
Aradu.T4VLF
Aradu.YRC2R
Arahy.0FWB0U
Arahy.K1SYDF
Arahy.LH2L98
Arahy.62AJ6F
Supplementary Figure S5. Expression patterns of several wild and cultivated peanut RWP-RK genes in five selected tissues. For genes in A. ipaensis, the expression level of Araip.R44NW in seedling leaves was set as 1, and the others were adjusted accordingly. For genes in A. duranensis, the expression level of Aradu.T4VLF in seedling leaves was set as 1, and the others were adjusted accordingly. For genes in A. hypogaea, the expression level of Arahy.0FWB0U in seedling leaves was set as 1, and the others were adjusted accordingly.

## Slide 6
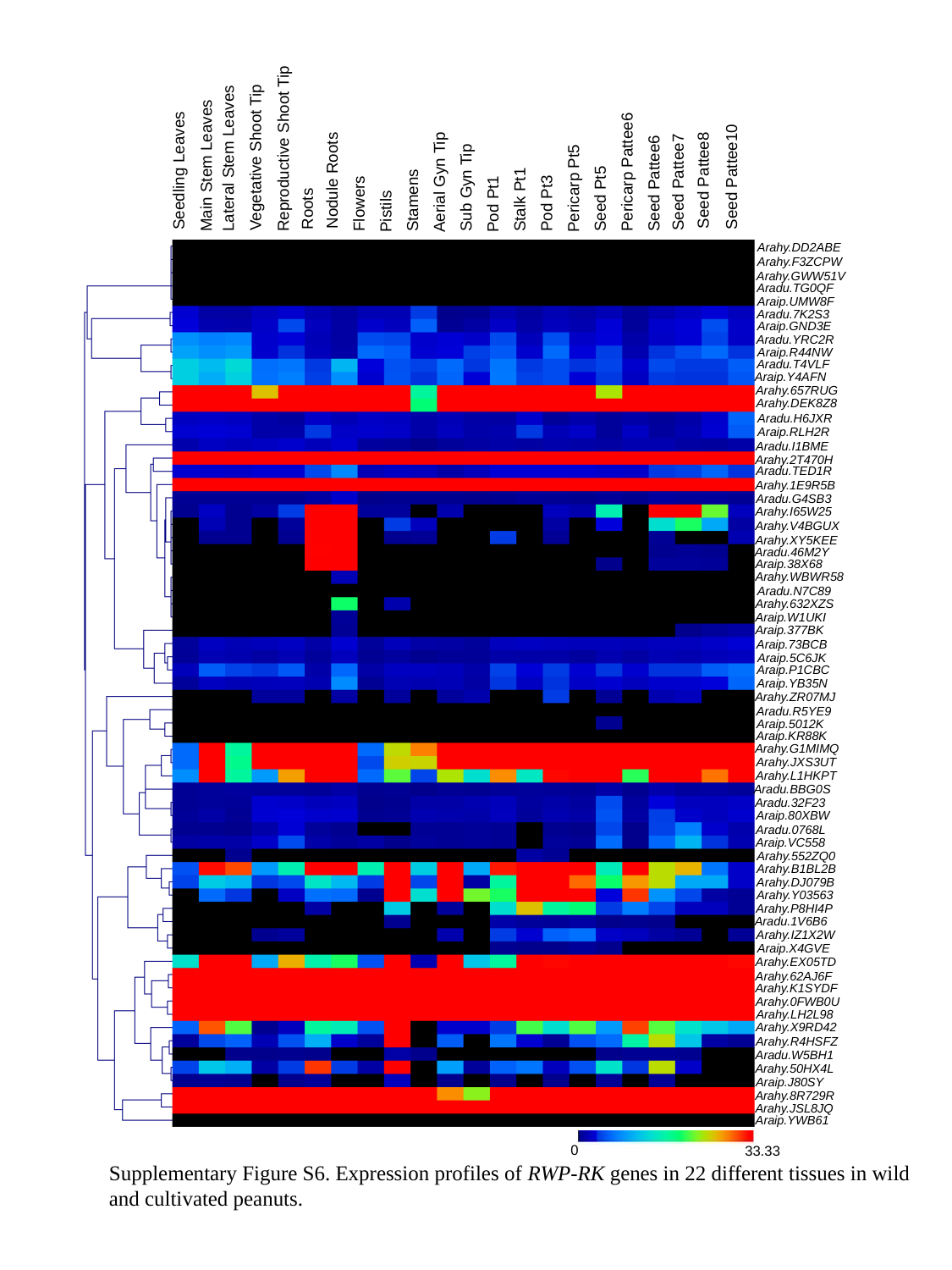

Nodule Roots
Stalk Pt1
Pod Pt1
Seed Pattee8
Seed Pattee10
Roots
Seedling Leaves
Vegetative Shoot Tip
Seed Pattee7
Reproductive Shoot Tip
Main Stem Leaves
Flowers
Stamens
Sub Gyn Tip
Pod Pt3
Seed Pt5
Pericarp Pattee6
Seed Pattee6
Lateral Stem Leaves
Pistils
Aerial Gyn Tip
Pericarp Pt5
Arahy.DD2ABE
Arahy.F3ZCPW
Arahy.GWW51V
Aradu.TG0QF
Araip.UMW8F
Aradu.7K2S3
Araip.GND3E
Aradu.YRC2R
Araip.R44NW
Aradu.T4VLF
Araip.Y4AFN
Arahy.657RUG
Arahy.DEK8Z8
Aradu.H6JXR
Araip.RLH2R
Aradu.I1BME
Arahy.2T470H
Aradu.TED1R
Arahy.1E9R5B
Aradu.G4SB3
Arahy.I65W25
Arahy.V4BGUX
Arahy.XY5KEE
Aradu.46M2Y
Araip.38X68
Arahy.WBWR58
Aradu.N7C89
Arahy.632XZS
Araip.W1UKI
Araip.377BK
Araip.73BCB
Araip.5C6JK
Araip.P1CBC
Araip.YB35N
Arahy.ZR07MJ
Aradu.R5YE9
Araip.5012K
Araip.KR88K
Arahy.G1MIMQ
Arahy.JXS3UT
Arahy.L1HKPT
Aradu.BBG0S
Aradu.32F23
Araip.80XBW
Aradu.0768L
Araip.VC558
Arahy.552ZQ0
Arahy.B1BL2B
Arahy.DJ079B
Arahy.Y03563
Arahy.P8HI4P
Aradu.1V6B6
Arahy.IZ1X2W
Araip.X4GVE
Arahy.EX05TD
Arahy.62AJ6F
Arahy.K1SYDF
Arahy.0FWB0U
Arahy.LH2L98
Arahy.X9RD42
Arahy.R4HSFZ
Aradu.W5BH1
Arahy.50HX4L
Araip.J80SY
Arahy.8R729R
Arahy.JSL8JQ
Araip.YWB61
0
33.33
Supplementary Figure S6. Expression profiles of RWP-RK genes in 22 different tissues in wild and cultivated peanuts.

## Slide 7
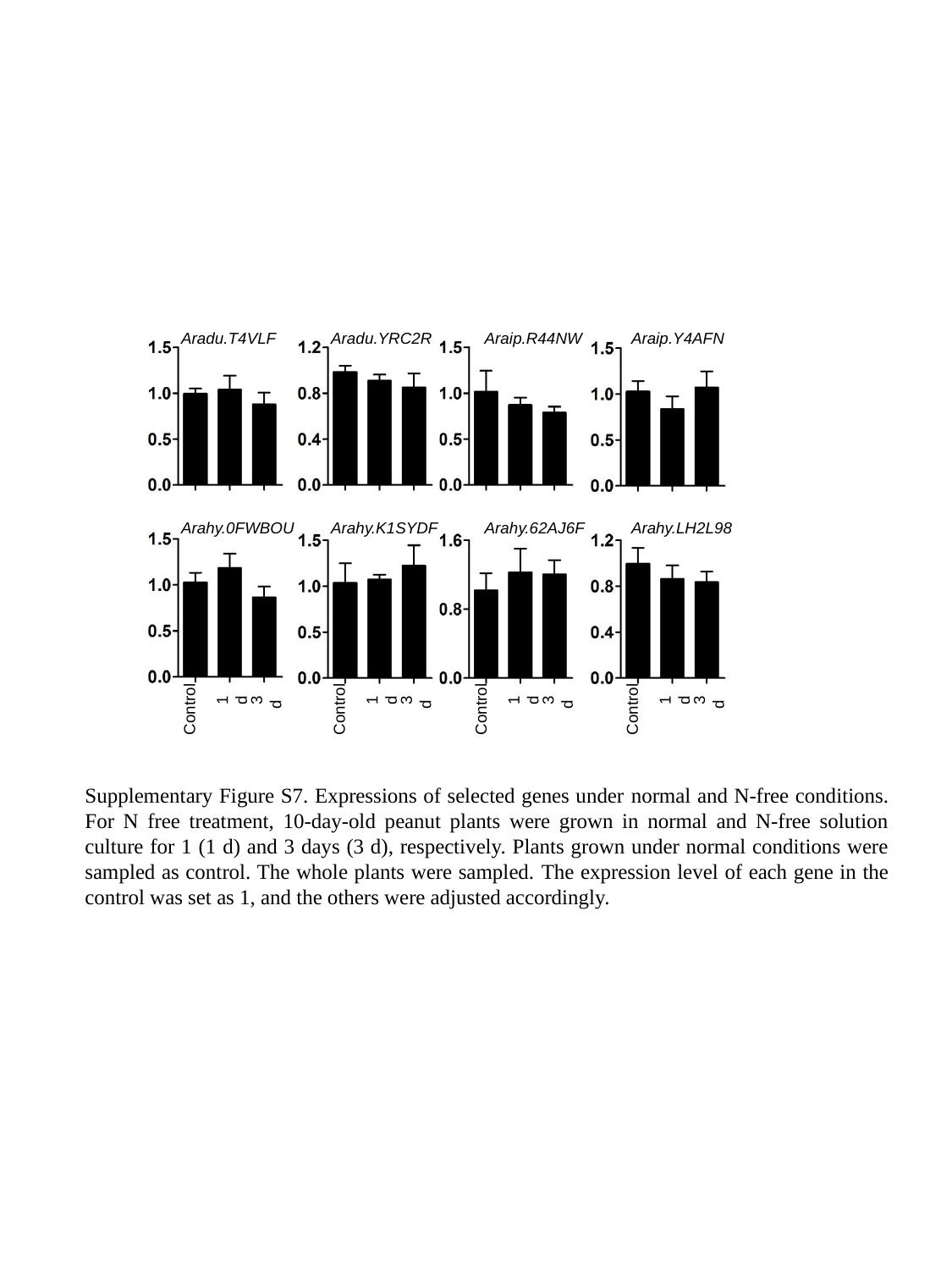

Aradu.T4VLF
Aradu.YRC2R
Araip.R44NW
Araip.Y4AFN
Arahy.0FWBOU
Arahy.K1SYDF
Arahy.62AJ6F
Arahy.LH2L98
Control
1 d
 3 d
Control
1 d
 3 d
Control
1 d
 3 d
Control
1 d
 3 d
Supplementary Figure S7. Expressions of selected genes under normal and N-free conditions. For N free treatment, 10-day-old peanut plants were grown in normal and N-free solution culture for 1 (1 d) and 3 days (3 d), respectively. Plants grown under normal conditions were sampled as control. The whole plants were sampled. The expression level of each gene in the control was set as 1, and the others were adjusted accordingly.
